# The transcriptomic (RNA-Sequencing) datasets collected in the course of floral induction in *Chenopodium ficifolium* 459

**DOI:** 10.1016/j.dib.2022.108333

**Published:** 2022-05-29

**Authors:** David Gutierrez-Larruscain, Manuela Krüger, Oushadee A.J. Abeyawardana, Claudia Belz, Petre I. Dobrev, Radomíra Vaňková, Kateřina Eliášová, Zuzana Vondráková, Miloslav Juříček, Helena Štorchová

**Affiliations:** Institute of Experimental Botany, Czech Academy of Sciences, Rozvojová 263, 16502 Prague, Czech Republic

**Keywords:** Flowering, Gene expression, Phytohormones, Oxidative stress, Photoperiod

## Abstract

The transition from vegetative growth to reproduction is the essential commitment in plant life. It is triggered by environmental cues (day length, temperature, nutrients) and regulated by the very complex signaling gene network and by phytohormones. The control of flowering is well understood in *Arabidopsis thaliana* and in some crops, much less is known about the other angiosperms. We performed the detailed transcriptomic survey of the course of floral induction in seedlings of *Chenopodium ficifolium* accession 459, a close relative of the important crop *Chenopodium quinoa*. It flowers earlier under short days (6 hours light) than under long days (18 hours light). Plants were sampled at the age 14, 18, 21 and 24 days in the morning and afternoon, both at long and short day, for RNA-Sequencing, and also for phytohormone analyses. We employed Illumina NovaSeq6000 platform to generate raw reads, which were cleaned and mapped against the *de novo* constructed transcriptome of *C. ficifolium*. The global gene expression levels between long and short days were pairwise compared at each time points. We identified differentially expressed genes associated with floral induction in *C. ficifolium* 459. Particular attention was paid to the genes responsible for phytohormone metabolism and signaling. The datasets produced by this project contributed to better understanding of the regulation of growth and development in the genus *Chenopodium*.

## Specifications Table

**Table:** 

Subject	Plant Science: General
Specific subject area	Transcriptomic changes during floral induction; differential gene expression under short and long photoperiod
Type of data	Table, graph, figure
How the data were acquired	Collection of seedlings grown under short and long days; RNA-Sequencing on Illumina NovaSeq6000 platform Software:TrimGalore, Trinity v.2.9.0, RSEM-EVAL, Salmon, DESeq2, in OmicsBox v.1.3.3.
Data format	Raw data: Illumina FASTQ files Analyzed data: tables, figures
Description of data collection	Above-ground parts of seedlings grown under long and short days were collected at the age 14, 18 21 and 24 days after sowing, in the morning and afternoon,16 time points were sampled altogether. Each time point was represented by three replicates, which generated 48 RNA specimens. The strand-specific cDNA libraries were prepared from 48 RNAs using polyA enrichment; sequencing produced 150 nt paired-end reads.
Data source location	Institute of Experimental Botany CAS Prague – Lysolaje Czech Republic 50°07′44″N 14°22′32 E
Data accessibility	Data can be accessed from NCBI SRA (BioProject ID: PRJNA771226) https://www.ncbi.nlm.nih.gov/bioproject/PRJNA771226 Graphs of Gene expression are available on Mendeley https://data.mendeley.com/datasets/gxh32vrrxc/2 DOI: 10.17632/gxh32vrrxc.2
Related research article	D. Gutierrez-Larruscain, M. Krüger, O.A.J. Abeyawardana, C. Belz, P.I. Dobrev, R. Vaňková, K. Eliášová, Z. Vondráková, M. Juříček, H. Štorchová. The High Concentrations of Abscisic, Jasmonic, and Salicylic Acids Produced Under Long Days Do Not Accelerate Flowering in Chenopodium Ficifolium 459, Plant Sci. 320 (2022) 111279. https://dx.doi.org/10.2139/ssrn.3994539.

## Value of the Data

• The gene expression data provide the comprehensive picture of transcriptomic changes during floral induction in *Chenopodium ficifolium* accession 459, making it possible to identify the genes, putatively involved in the regulation of flowering.
• The transcriptomic data set may be used not only by the specialists investigating flowering, but also by numerous researchers interested in plant growth and development, plant stress response and phytohormone function.
• This comprehensive data set may be also used for the comparison with the course of floral induction in *C. ficifolium* accessions with the opposite response to photoperiod, which flower earlier under long days, or for the comparison with the important crop *Chenopodium quinoa*. The integrative analysis of transcriptomic and hormonomic data will contribute to the creation of the plausible model of the control of flowering in the genus *Chenopodium*, which is phylogenetically distant from the current model plants.

## 1 Data Description

The general overview of the transcriptomic data is given in Table 1, which presents the accession numbers of raw data generated by RNA sequencing at particular time points of the floral induction experiment, as well as the counts of raw and trimmed Illumina reads. Clean reads were mapped against the reference *de novo* transcriptome of *C. ficifolium* by Salmon and differential expression (DE) between short day (SD)-treated and long day (LD)-treated plants in particular time points was estimated by DESeq2. The most highly DE genes were analyzed for GO enrichment by OmicsBox v.1.3.3. Table 2 shows the enriched GO categories among 6096 DE genes, with the sum of log2fold above a selected threshold. GO categories include hydrogen peroxide catabolism, hydrolase and peroxidase activities, or defense response.

**Table 1:** Accession numbers and read counts for raw data of the transcriptomes from the specific time points in the course of floral induction (days after sowing, DAS) in *C. ficifolium* 459 under short and long days.

	SRA Acc.	BioSample	Raw reads	Clean reads
Sample	number	Acc. number	Count	Count
14 DAS, long day, 9h, replicate 1	SRR16327180	SAMN22258499	31667200	25237264
14 DAS, long day, 9h, replicate 2	SRR16327179	SAMN22258499	31580742	23570100
14 DAS, long day, 9h,replicate 3	SRR16327168	SAMN22258499	31227038	24088022
14 DAS, short day, 9h, replicate 1	SRR16327157	SAMN22258497	31763692	24011046
14 DAS, short day, 9h, replicate 2	SRR16327146	SAMN22258497	30977148	23364624
14 DAS, short day, 9h, replicate 3	SRR16327137	SAMN22258497	31672624	24327898
14 DAS, long day, 15h, replicate 1	SRR16327136	SAMN22258500	31845692	23117646
14 DAS, long day, 15h, replicate 2	SRR16327135	SAMN22258500	31443614	23654190
14 DAS, long day, 15h, replicate 3	SRR16327134	SAMN22258500	31502474	23496338
14 DAS, short day, 15h, replicate 1	SRR16327133	SAMN22258498	30706270	23474004
14 DAS, short day, 15h, replicate 2	SRR16327178	SAMN22258498	30915796	21226688
14 DAS, short day, 15h, replicate 3	SRR16327177	SAMN22258498	31690760	23863816
18 DAS, long day, 9h, replicate 1	SRR16327176	SAMN22258499	31231276	21603854
18 DAS, long day, 9h, replicate 2	SRR16327175	SAMN22258499	31133282	22193808
18 DAS, long day, 9h,replicate 3	SRR16327174	SAMN22258499	31633238	23632230
18 DAS, short day, 9h, replicate 1	SRR16327173	SAMN22258497	31575898	23627814
18 DAS, short day, 9h, replicate 2	SRR16327172	SAMN22258497	31676814	23512640
18 DAS, short day, 9h, replicate 3	SRR16327171	SAMN22258497	31075172	22072280
18 DAS, long day, 15h, replicate 1	SRR16327170	SAMN22258500	31287418	23104260
18 DAS, long day, 15h, replicate 2	SRR16327169	SAMN22258500	31656892	24038868
18 DAS, long day, 15h, replicate 3	SRR16327167	SAMN22258500	31694468	23785548
18 DAS, short day, 15h, replicate 1	SRR16327166	SAMN22258498	31436216	23472138
18 DAS, short day, 15h, replicate 2	SRR16327165	SAMN22258498	31879318	24211942
18 DAS, short day, 15h, replicate 3	SRR16327164	SAMN22258498	31300048	21720620
21 DAS, long day, 9h, replicate 1	SRR16327163	SAMN22258499	31574384	24096876
21 DAS, long day, 9h, replicate 2	SRR16327162	SAMN22258499	30761014	22897816
21 DAS, long day, 9h,replicate 3	SRR16327161	SAMN22258499	31503612	23672998
21 DAS, short day, 9h, replicate 1	SRR16327160	SAMN22258497	31286494	22886510
21 DAS, short day, 9h, replicate 2	SRR16327159	SAMN22258497	30914284	27064858
21 DAS, short day, 9h, replicate 3	SRR16327158	SAMN22258497	30843778	22724770
21 DAS, long day, 15h, replicate 1	SRR16327156	SAMN22258500	31733782	23085602
21 DAS, long day, 15h, replicate 2	SRR16327155	SAMN22258500	31758884	26612024
21 DAS, long day, 15h, replicate 3	SRR16327154	SAMN22258500	31808934	24140268
21 DAS, short day, 15h, replicate 1	SRR16327153	SAMN22258498	31802238	24314844
21 DAS, short day, 15h, replicate 2	SRR16327152	SAMN22258498	30700020	23692024
21 DAS, short day, 15h, replicate 3	SRR16327151	SAMN22258498	30935584	24828554
24 DAS, long day, 9h, replicate 1	SRR16327150	SAMN22258499	31720020	23534488
24 DAS, long day, 9h, replicate 2	SRR16327149	SAMN22258499	31881212	24172540
24 DAS, long day, 9h,replicate 3	SRR16327148	SAMN22258499	31680512	23677028
24 DAS, short day, 9h, replicate 1	SRR16327147	SAMN22258497	31262218	23541728
24 DAS, short day, 9h, replicate 2	SRR16327145	SAMN22258497	31292960	23814588
24 DAS, short day, 9h, replicate 3	SRR16327144	SAMN22258497	31508030	22978662
24 DAS, long day, 15h, replicate 1	SRR16327143	SAMN22258500	31077372	22750554
24 DAS, long day, 15h, replicate 2	SRR16327142	SAMN22258500	31272044	25127910
24 DAS, long day, 15h, replicate 3	SRR16327141	SAMN22258500	30725606	23050154
24 DAS, short day, 15h, replicate 1	SRR16327140	SAMN22258498	31483114	23939406
24 DAS, short day, 15h, replicate 2	SRR16327139	SAMN22258498	31008196	24411794
24 DAS, short day, 15h, replicate 3	SRR16327138	SAMN22258498	30932056	23099512
Flowers, ambient conditions	SRR19142492	SAMN28159737	31578462	22539338
Leaves, ambient conditions	SRR19142491	SAMN28159737	31680494	22715818
Roots, ambient conditions	SRR19142490	SAMN28159737	31204052	23618816

**Table 2:** Enriched GO terms (False Discovery Rate (FDR) < 0.05) among 6096 differentially expressed (DE) genes between short day- and long day-treated *C. ficifolium* 459. The number of DE genes (with log fold change summed values across time points above the threshold of 10) related to the enriched GO terms (BP – Biological Process, CC – Cellular Component, MC – Molecular Function) are shown as counts with their respective p-value and FDR.

GO ID	GO Term	GOCategory	FDR	p-value	Count
GO:0042744	hydrogen peroxide catabolic process	BP	2.51E-05	2.80E-08	35
GO:0009694	jasmonic acid metabolic process	BP	0.001037	2.32E-06	10
GO:0006952	defense response	BP	0.001182	2.86E-06	53
GO:0044550	secondary metabolite biosynthetic process	BP	0.010539	3.34E-05	14
GO:0009834	plant-type secondary cell wall biogenesis	BP	0.026001	9.44E-05	11
GO:0045492	xylan biosynthetic process	BP	0.026001	9.44E-05	11
GO:1990748	cellular detoxification	BP	0.040678	1.67E-04	44
GO:0009813	flavonoid biosynthetic process	BP	0.040748	1.74E-04	6
GO:0048046	apoplast	CC	5.10E-04	9.50E-07	34
GO:0009505	plant-type cell wall	CC	0.01824	6.11E-05	23
GO:0005886	plasma membrane	CC	0.035955	1.41E-04	178
GO:0020037	heme binding	MF	2.83E-09	7.91E-13	84
GO:0003700	DNA-binding transcription factor activity	MF	3.68E-06	1.37E-09	97
GO:0016705	oxidoreductase activity, acting on paired donors, with incorporation or reduction of molecular oxygen	MF	7.52E-06	4.90E-09	62
GO:0004497	monooxygenase activity	MF	1.54E-04	2.58E-07	51
GO:0004553	hydrolase activity, hydrolyzing O-glycosyl compounds	MF	5.61E-04	1.10E-06	78
GO:0005506	iron ion binding	MF	6.26E-04	1.28E-06	55
GO:0010333	terpene synthase activity	MF	0.003772	9.83E-06	7
GO:0004601	peroxidase activity	MF	0.005245	1.47E-05	39
GO:0016762	xyloglucan:xyloglucosyl transferase activity	MF	0.01824	6.10E-05	11
GO:0080043	quercetin 3-O-glucosyltransferase activity	MF	0.029037	1.11E-04	13
GO:0080044	quercetin 7-O-glucosyltransferase activity	MF	0.029037	1.11E-04	13

**Fig. 1:**
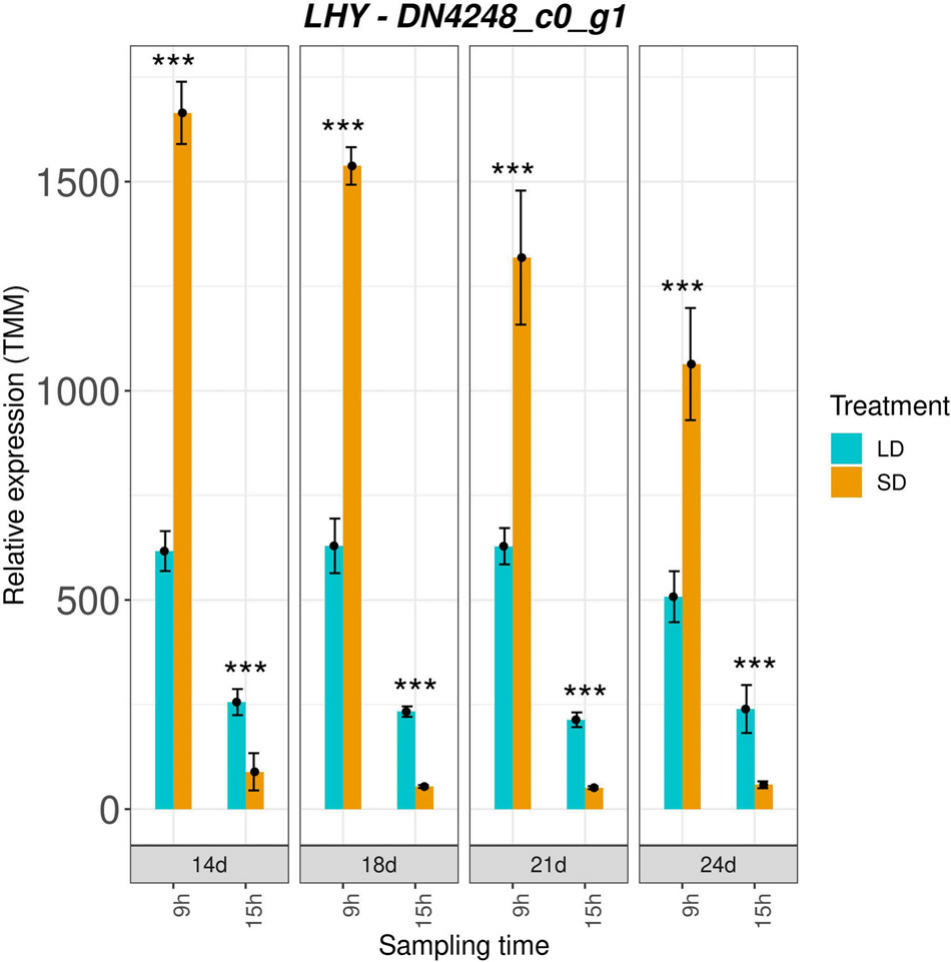
The expression of the *LATE ELONGATED HYPOCOTYL* (*LHY*) homolog in *C. ficifolium* 459 at the age 14, 18 21, and 24 days after sowing under long and short days. Blue columns correspond to LD treated samples, golden ones represent SD treated samples. Transverse lines at each dot (median value of three biological replicates) represent standard deviation. Statistical significance (p values * < 0.05, ** < 0.01 and ***< 0.001; estimated by DESeq2; three biological replicates, each consisting of 3 to 5 seedlings) between pairs of differentially treated samples is represented by asterisks. The x-axis represents eight sampling points (two sampling points per day: morning - 9.00, and afternoon -15.00). The y-axis represents relative expression in transcript coverage as trimmed mean of M-values (TMM).

We generated the graphs of gene expression in the course floral induction under contrasting photoperiods. Fig. 1 shows the graph for the *LATE ELONGATED HYPOCOTYL* (*LHY*) gene as an example. *LHY* is the homolog of the central clock oscillator gene in *A. thaliana* and might have performed the same function in *C. ficifolium*, too.The gene expression graphs for the phytohormone-related genes, which were not presented in [1] are accessible on Mendeley (DOI: 10.17632/gxh32vrrxc.2).The graphs were constructed from TMM coverage values and log2 fold changes between SD- and LD-grown plants.

## 2 Experimental Design, Materials and Methods

### 2.1 Plant material

The accession *C. ficifolium* 459 was originally collected in Central Asia [2]. The plants were cultivated in the Institute of Experimental Botany greenhouse and propagated by self-pollination. Seeds were surface-sterilized and germinated as described by Štorchová et al. [2]. Average-sized seedlings with opened cotyledons and uniform growth were selected for the experiments. Plants planted in 96-well flat-bottom ELISA plates, single seedling per well, soaked in half-strength Hoagland solution, were maintained under 22 °C, 70% humidity, and cool-white fluorescent light (130 μmol m^−2^ s^−1^) or dark in growth chamber Percival E-36L2. Two contrasting photoperiodic regimes were applied: SD (6 h light and 18 h dark) and LD (18 h light and 6 h dark) for the floral induction analysis.

Growth analyses started using vegetative seedlings ten days after sowing (DAS). Measurements were made five times in the interval of 4-5 days (until flowering). Usually six plants from each treatment were used. The images of the whole seedlings, isolated cotyledons and leaves placed into the Petri dishes were examined under Navitar Machine Vision (Navitar Inc., Rochester, NY, USA). The length of shoot apex and flowering rate were assessed under a stereomicroscope Zeiss Stemi 305. The rate of flowering was stated as the number of plants with terminal flower bud (in % from the whole set of tested plants). All plants cultivated under SD formed flower buds at the age 24 DAS, compared with only 20% of flowering plants grown under LD. All LD cultivated plants reached the flowering stage at 32 DAS.

### 2.2 RNA sampling and extraction

The seedlings were collected twice a day (in the morning at 9.00 and the afternoon at 15.00) at 14, 18, 21 and 24 DAS under SD and LD. The light was switched on at 9.00 under both regimes. Above-ground parts of the seedlings (14 and 18 DAS) or upper leaves and stems with apical parts of young plants (21 and 24 DAS) from each photoperiodic regime were collected and flash-frozen in liquid nitrogen. Three biological replicates, each consisting of three to four seedlings from LD conditions and eight to ten seedlings from SD conditions, were sampled at each time point. Total RNA was extracted using a Plant RNeasy Mini kit (Qiagen, Valencia, CA, USA). DNase I treatment was performed according to the manufacturer‘s protocol (DNA-free, Ambion, Austin, TX, USA) to remove genomic DNA. If necessary, the DNase I treatment was done twice to eliminate any traces of genomic DNA. RNA concentration and quality were checked on 0.9% agarose gel and using the NanoDrop (Thermo Fisher Scientific, Vantaa, Finland).

### 2.3 RNA-Sequencing

Total RNAs extracted from the seedlings collected at eight time points under SD and LD were stabilized by GenTegra technology (GenTegra, Pleasanton, California, USA) and sent to Macrogen (Seoul, Korea) in GenTegra microtubes. Strand-specific cDNA libraries were constructed from polyA enriched RNA. Additional RNAs were prepared from leaves, flowers, and roots of mature plants grown in the greenhouse to supplement seedling RNA specimens to achieve the more complete transcriptome assembly. Strand-specific cDNA libraries were constructed from polyA enriched RNA and sequenced on the Illumina NovaSeq6000 platform.

**Fig. 2:**
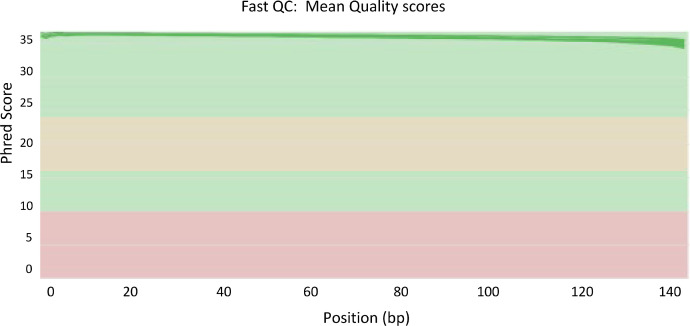
The quality metrics in phred scores per base (raw fastq reads) for the transcriptome of *Chenopodium ficifolium* 459 deposited in the SRA database under the accession number PRJNA771226.

We obtained 753,019,719 paired-end (PE) reads (150 nt), about 14.8 million reads per sample. The read quality in phred scores per base is shown in Fig. 2. These raw reads were first error corrected using Rcorrector [3] with default settings, to address random sequencing errors in the RNA-Seq dataset.

After error correction, ribosomal RNA was filtered out deploying SortMeRNA [4] using the provided silva rRNA databases as reference. The resulting sequencing reads were further quality and adapter trimming with TrimGalore [5]. Here, we used the trimming lengths of 145 bp with quality trimming (-q) of 5, for stringency and maximum allowed error rates default options (–stringency 1, -e 0.1). This trimming procedure removed approximately 25% of the data, leaving 567,261,573 paired-reads after the cutoff.

The raw and trimmed reads of the 48 samples (14, 18, 21, and 24 DAS) were deposited under the BioProject number PRJNA771226 with SRA accessions SRR16327138-SRR16327180 for the raw reads and SRR16380491-SRR16380533 for the trimmed reads. The raw and trimmed reads of three samples (leaves, roots, and flowers of adult plants are available under the same BioProject number under SRA accessions SRR19142490-SRR19142492, and SRR19143407- SRR19143409, respectively.

### 2.4 Transcriptome assembly and evaluation

Part of the trimmed reads, one replicate per sampling time point and treatment, as well as the three individual samples from leaves, roots and flowers of adult plants, were used for the *de novo* assembly with Trinity v.2.9.0 [6] with default options and the strand-specific RNA-Seq read orientation parameter (–SS_lib_type RF). The resulting assembly was first roughly evaluated with the perl script within the Trinity pipeline (StatsTrinity.pl) resulting in 213,741 transcripts and 168,036 potential ‘genes’, and an N50 value of 1530 based on all transcripts.

The redundancy of the Trinity assembly was first reduced with CD-Hit v.4.8.1 [7] applying a similarity cutoff of 99.9%. It was followed by a step, which resulted in a more condensed and non-redundant transcript assembly, with the script EvidentialGene tr2aacds.pl using MINCDS = 50. The resulting okay set, containing 55,020 transcripts and 51,146 potential genes, was used as the final assembly and input for a blastx search against the nr database. The blastx results were obtained using the command line application with the faster blastx-fast version. The parameters employed for the blastx search against the nr-database were an e-value of 0.01 and a maximum of 10 target sequences. The BLASTX results were imported into the MEGAN pipeline [8], with only plant hits retained.

The evigene assembly was used for all subsequent analyses and deposited at DDBJ/ENA/GenBank in the TSA archive under the accession GJOD01000000. The version described in this paper is the first version, GJOD01000000.

After this step, we applied three evaluation methods to check the quality of the assembly. First we used BUSCO v.3.1.0. [9] with the embryophytes_odb9 database and in transcriptome mode (–mode trans) to access the assembly. BUSCO reported 1329 complete, from which 1279 are single copy and 50 duplicated, 34 fragmented and 76 missing BUSCOs. Second we employed detonate with the RSEM-EVAL package v.1.11 [10] using bowtie2 with the transcript-length-parameters 959_APVO_SCC_Genes.fasta, as true_transcript_length_distribution, the –strand-specific and –paired-end option for the 145 bp reads assembly. This evaluation resulted in a score of -78578280472.09. Finally, a custom script was used to evaluate the completeness and contiguity of the Trinity assembly as described in [11]. The assembly showed a completeness of 0.915 and contiguity of 0.904.

To annotate the transcriptome, blastx-based homology searches (BLAST + 2.9.0) for the final transcriptome assembly against the NCBI nr protein database were performed. The cutoff E-value was set to <10-4, and the maximum number of allowed hits was set to 10. The OmicsBox program v. 1.3.3 (BioBam Bioinformatics S.L., Valencia, Spain) was then used to annotate the “Trinity” genes based on gene ontology (GO) terms, InterProScan, and nr database annotation.

### 2.5 Transcript quantification and pairwise differential expression

Transcript quantification was done with the Trinity pipeline, using the alignment-free method Salmon v.1.4.0 [12] with default parameter, but specifying the single stranded library with –SS_lib_type RF for all samples (48) at each sampling time point. The resulting estimated fragment counts and normalized expression metrics (transcripts per million transcripts; TPM) were reported for the transcripts and trinity ‘genes’ in each of the samples. In the next step a matrix of estimated counts and a second matrix of cross-sample normalized expression values using the TMM (trimmed mean of M-values) method was built for all samples on the transcript and gene-level. These matrices were used for the subsequent analyses of DE genes.

The differential gene expression analysis was carried out on both, the transcript and trinity ‘gene’ level, using the Bioconductor package DESeq2 v.1.32.0 [13] and the scripts within the Trinity pipeline. The three biological replicates for each sampling time point were pairwise compared contrasting the LD with the SD condition. The standard single time point analysis was used. Extraction of DE genes was done for each sampling time point with 0.05 cutoff for corrected FDR p-values. For the subsequent analyses only gene-level data was used.

To set the collection of DE genes used for the Gene Ontology Term Enrichment analysis (GO analysis), an index was created based on the Fold Change values between SD and LD treated samples obtained through the software DESeq2 [13]. Absolute values of log2 Fold Change for each DE gene between SD and LD at each sampling time point were summed up. High values of the sum denoted high pair differences in the expression between SD and LD, both positive and negative. The thresholds of 10, 15, and 20 index sum values corresponding to 6096, 3011, and 1545 DE genes, respectively, were selected to perform GO analysis. After comparing the GO analysis outputs and the gene expression graphs of selected DE genes, the set of 6096 genes was chosen as the most robust set for the GO enrichment analysis. The Fisher exact test (p-value < 0.05) implemented in OmicsBox program v. 1.3.3 was utilized for this analysis.

## Ethics Statements

Our data was obtained from plant material, no animals were used.

## CRediT Author Statement

**David Gutierrez-Larruscain:** Software, Writing, Visualization; **Manuela Krüger:** Software, Formal Analysis, Writing; **Oushadee A.J. Abeyawardana:** Data curation, Methodology; **Claudia Belz:** Data curation, Methodology; **Petre I. Dobrev:** Data curation, Methodology; **Radomíra Vaňková:** Validation, Investigation; **Kateřina Eliášová:** Data curation, Validation; **Zuzana Vondráková:** Data curation, Validation; **Miloslav Juříček:** Software, Formal Analysis; **Helena Štorchová:** Conceptualization, Funding acquisition, Writing, Supervision.

## Declaration of Competing Interest

The authors declare that they have no known competing financial interests or personal relationships that could have appeared to influence the work reported in this paper.

## Data Availability

Repository of Supplementary files from the paper entitled: “The transcriptomic (RNA-Sequencing) datasets collected in the course of floral induction in Chenopodium ficifolium 459” (Original data) (Mendeley Data).
